# Motivators and Barriers to COVID-19 Vaccination Intentions Across U.S. County-Level Barriers in the COVID-19 Vaccine Coverage Index

**DOI:** 10.1007/s40615-024-02096-9

**Published:** 2024-08-02

**Authors:** Jessica R. Fernandez, Jennifer Richmond, Paula D. Strassle, Jennifer Cunningham-Erves, Allana T. Forde

**Affiliations:** 1 Division of Intramural Research, National Institute On Minority Health and Health Disparities, National Institutes of Health, Bethesda, MD USA; 2 Department of Social Sciences and Health Policy, Wake Forest University School of Medicine, Winston-Salem, NC USA; 3 Department of Health Policy, Vanderbilt University Medical Center, Nashville, TN USA

**Keywords:** Motivators and barriers to COVID-19 vaccination intentions, Health disparities, Race/ethnicity, Structural barriers, County-level vaccination barriers, COVID-19 preventive health services

## Abstract

**Background:**

County-level barriers (sociodemographic barriers, limited healthcare system resources, healthcare accessibility barriers, irregular healthcare seeking behaviors, low vaccination history) may impact individuals’ reasons for receiving the COVID-19 vaccine.

**Methods:**

This study linked data from REACH-US (Race-Related Experiences Associated with COVID-19 and Health in the United States), a nationally representative, online survey of 5475 adults living in the U.S (January-March 2021) to county-level barriers in the COVID-19 Vaccine Coverage Index. County-level vaccination barriers were measured using the COVID-19 Vaccine Coverage Index. Participants reported why they would or would not receive the COVID-19 vaccine in an open-ended item and their responses were coded using thematic analysis. Descriptive statistics and chi-square tests assessed whether reasons for COVID-19 vaccination intentions varied by county-level barriers and whether these distributions varied across racial/ethnic groups.

**Results:**

Thematic analysis revealed twelve themes in participants’ reasons why they would or would not receive the COVID-19 vaccine. Themes of societal responsibility (9.8% versus 7.7%), desire to return to normal (8.1% versus 4.7%), and trust in science/healthcare/government (7.7% versus 5.1%) were more frequently reported in counties with low/medium barriers (versus high/very high) (p-values < 0.05). Concerns of COVID-19 vaccine side effects/safety/development (25.3% versus 27.9%) and concerns of access/costs/availability/convenience (1.9% versus 3.6%) were less frequently reported in counties with low/medium barriers (versus high/very high) (p-values < 0.05). Trends in the prevalence of these themes varied across racial/ethnic groups (p-values < 0.05).

**Conclusions:**

Future pandemic responses should consider potential ways county-level barriers shape reasons for COVID-19 vaccination.

**Supplementary Information:**

The online version contains supplementary material available at 10.1007/s40615-024-02096-9.

The United States (U.S.) approved emergency use authorization for three vaccines to protect against the SARS-COV-2 virus (i.e., the virus that caused the coronavirus disease 2019 [COVID-19]) between December 2020 and March 2021 [1]. COVID-19 vaccines were initially available to adults who worked and/or lived in health care or long-term care settings, were older than 65 years of age, had high-risk medical conditions, or were essential workers at high-risk of exposure to the SARS-COV-2 virus [2]. COVID-19 vaccines were made available to all individuals ≥ 16 years of age in the U.S. by April 19, 2021 [1]. However, during the initial stages of the U.S. vaccination program, many adults did not receive the COVID-19 vaccine and racial/ethnic disparities in uptake were observed between December 2020–November 2021 [3]. Particularly, American Indian/Alaska Native, Black/African American, Hispanic/Latino, Multiracial, and Native Hawaiian/Pacific Islander adults had lower vaccination rates compared to Asian and White adults in the U.S. [3].

Studies conducted in the early stages of the COVID-19 vaccine rollout (i.e., November 2020–March 2021) examined reasons why adults would or would not receive the COVID-19 vaccine (i.e., “COVID-19 vaccination intentions”) [4–7]. A large, national survey of U.S. adults conducted from February 16–21, 2021 found that reasons associated with COVID-19 vaccine receipt included trust in vaccine research and development, considerations of community health, and personal concerns of developing serious COVID-19 disease [7]. Alternatively, multiple studies conducted during similar timeframes found that adults reported not receiving the COVID-19 vaccine due to complacency (e.g., vaccine unnecessary; low concern for COVID-19), poor convenience (e.g., costs and access), and/or low vaccine confidence (e.g., mistrust in vaccines, vaccine researchers and manufacturers, and/or the healthcare system; perceived side effects and/or rushed development) [4–8]. These concerns often varied across racial/ethnic groups [9] and reflected historical structural racism, a major driver of inequities in the U.S. healthcare system (e.g., racial/ethnic disparities in access to healthcare, healthcare discrimination) and/or historical exploitation in biomedical studies [10, 11]). Concerns about COVID-19 vaccination side effects, for example, were higher among Non-Hispanic Black adults compared to Non-Hispanic White adults and concerns about costs, access to, and/or the rushed development of the COVID-19 vaccine were higher among Hispanic and Non-Hispanic Black adults compared to White adults [12–14]).

Given the relationship between COVID-19 vaccination intentions and structural healthcare inequities [15], recent studies examined whether county-level factors influenced individuals’ COVID-19 vaccination intentions [16]. Social vulnerabilities, for example, were assessed in a tool developed by the Centers for Disease Control and Prevention (CDC) (i.e., social vulnerabilities measured by socioeconomic status, household composition and disability, racial/ethnic minority status and language, housing type, and transportation) [17, 18]. Those living in counties with higher social vulnerabilities were more likely to report greater COVID-19 vaccine hesitancy [19, 20]. More recently, the CDC released the COVID-19 Vaccine Coverage Index (CVAC index) created by Surgo Ventures [21]. The CVAC index includes U.S. county-level scores of social vulnerabilities (i.e., socioeconomic disadvantage and lack of access to information), healthcare barriers (i.e., limited healthcare system resources, healthcare accessibility barriers, and irregular healthcare seeking behaviors), and low vaccination history. A recent study found that CVAC scores varied across racial/ethnic groups, were associated with COVID-19 vaccination intentions, and the associations between county-level barriers varied across racial/ethnic groups [22].

Despite the large body of evidence on COVID-19 vaccination intentions, there are gaps in the literature. COVID-19 vaccination intentions are complex and there are different reasons why participants would or would not receive the vaccine [9, 23]. Yet, many previous studies of the U.S. population focused on reasons why participants were unsure or unwilling to receive the COVID-19 vaccine. Fewer studies examined reasons why individuals would choose to vaccinate [24]. In addition, most studies used close-ended formats [23]. Although informative, close-ended formats relied on fixed-choice responses generated by the researcher(s) that could potentially leave out important factors related to individuals’ intentions [25–27]. Open-ended formats (e.g. text entry boxes allowing participants to answer questions in their own words) can generate a more diverse response set that might not be captured using fixed-choices [26]. Lastly, in studies that used open-ended formats to capture reasons for COVID-19 vaccination intentions, there was a lack of emphasis on how these reasons varied by structural factors such as county-level barriers and whether these differences varied across racial/ethnic groups. This is critical given that vaccine hesitancy is context-specific [8], and these findings can yield more targeted strategies by geographical location.

The present mixed-method study linked quantitative CVAC scores with qualitative open-ended reasons for COVID-19 vaccination intentions collected from a large, diverse population of adults living in the U.S. The study aimed to 1) identify themes in the open-ended reasons for why participants would or would `not receive the COVID-19 vaccine, 2) examine whether themes in open-ended reasons for COVID-19 vaccination intentions varied by county-level barriers, and 3) examine whether these distributions varied across racial/ethnic groups.

## Methods

### Data Source

This study used data from the Race-Related Experiences Associated with COVID-19 and Health in the United States (REACH-US) study. County-level barriers (see *Measures*) were obtained from the publicly available CVAC index and linked with data from the REACH-US study using the U.S. ZIP Code reported by REACH-US study participants.

#### REACH-US Study

The REACH-US study was a cross-sectional, online survey of 5,500 adults from seven racial/ethnic subgroups (i.e., 500 American Indian or Alaska Native, 1000 Asian, 1000 Black or African American, 1000 Hispanic or Latino, 500 Multiracial, 500 Native Hawaiian or Pacific Islander, and 1000 White adults) living in the U.S. Study participants answered survey items about their health, receipt of COVID-19 testing and vaccination, attitudes about the COVID-19 vaccine, perceptions about COVID-19 healthcare, and other items about race-related stressors and life experiences. Participants were compensated for their time.

Participants were recruited from an existing opt-in survey panel hosted by YouGov, a nonpartisan research firm. Participants were proximity matched to a target sample of U.S. adults generated from the 2018 American Community Survey 1-year data based on race/ethnicity, gender, age, education level, and language preference (English Language Preference or Spanish Language Preference for the Hispanic/Latino subgroup only). Responses were collected from participants between January 26, 2021 and March 3, 2021. Additional details including the recruitment methods, the stratified sampling approach to include a diverse set of racial/ethnic groups, the matching process, and propensity scoring to generate sample weights and nationally representative estimates within racial/ethnic groups is published elsewhere [22]. The quota sampling method, along with the matching and propensity scoring were implemented to increase diversity and facilitate comparisons across racial/ethnic groups and to potentially mitigate issues related to sampling biases.

The final sample included in the present study was 5,499 participants, after excluding one participant with a missing ZIP Code. This study was determined to be exempt, non-human subjects research by the National Institutes of Health Institutional Review Board given that YouGov provided deidentified data to the study team. All study participants were ≥ 18 years of age.

### Measures

#### County-Level Barriers

County-level barriers were obtained from the publicly available CVAC index (details on the CVAC index can be found elsewhere [21]). The CVAC index includes a set of five vaccination barriers for each county in the U.S: sociodemographic barriers (i.e., socioeconomic disadvantage, lack of access to information), limited healthcare system resources (i.e., healthcare system capacity, quality and spending of healthcare resources), healthcare accessibility barriers (i.e., cost barriers, transportation barriers), irregular care-seeking behavior (i.e., lack of designated medical home, lack of routine care visits), and low vaccination history (i.e., lower coverage and high refusal rates of various vaccines).

The CVAC index includes a composite score and individual scores of the five vaccination barriers (sociodemographic barriers, limited healthcare system resources, healthcare accessibility barriers, irregular care-seeking behavior, low vaccination history). Missing data values were imputed with median values across all counties. Stepwise percentile ranking and equal weighting of the five barriers were used to calculate the composite CVAC scores [21].

The present study used the composite CVAC score (i.e., “county-level barriers”). Scores ranged from 0 to 1, with higher scores representing greater barriers. Consistent with the original coding outlined in the CVAC Methodology report [21], the analysis categorized counties with a barrier score ≤ 0.6 as “low/medium” and counties with a barrier score > 0.6 as “high/very high.”

#### Open-Ended Reasons Why Participants Would or Would Not Receive the COVID-19 Vaccine

Participants were asked “Please indicate why you would or would NOT get the vaccine when it becomes available?”. The response option included an open text box for their answer.

#### Racial/Ethnic Group

Participants self-identified whether they were of Hispanic, Latino, or Spanish origin (i.e., “No” or “Yes”). Participants who selected “Yes” were included in a subgroup labeled “Hispanic/Latino” which included participants from all gender identities. Participants in the Hispanic/Latino subgroup then selected their language preference for the survey (i.e., English or Spanish). Lastly, participants self-identified their race from the 2020 U.S. Census categories. The ethnicity, language preference, and race items were combined into the categories of American Indian/Alaska Native, Asian, Black/African American, Hispanic/Latino English Language Preference (ELP), Hispanic/Latino Spanish Language Preference (SLP), Multiracial, Native Hawaiian/Pacific Islander, and White.

### Data Analysis

Descriptive statistics and chi-square tests were used to assess the frequency distributions of selected sociodemographic characteristics of the study population and county-level barriers, overall and stratified by racial/ethnic group.

A mixed-method approach was performed to address the study objectives. First, qualitative analysis was used to identify themes in the open-ended reasons for why participants would or would not receive the COVID-19 vaccine (“themes in COVID-19 vaccination intentions”). Second, qualitative and quantitative data were integrated through data transformation [28]. The presence and absence of qualitative themes in COVID-19 vaccination intentions were transformed into quantitative binary variables and merged with the CVAC county-level barriers data. Third, quantitative analysis was used to assess whether themes in COVID-19 vaccination intentions varied by participants’ county-level barriers and whether there were racial/ethnic differences in the themes stratified by county-level barriers. This mixed-method analysis process is described in more detail below.

#### Themes in COVID-19 Vaccination Intentions

Two analysts on the research team (JRF and JR) used an iterative inductive/deductive approach to develop a codebook [29–31]. In the inductive stage, the analysts reviewed all data to create codes identified from participants’ responses. In the deductive stage, the analysts organized codes into themes informed by the vaccination decision literature [32–34]. This approach allowed for drawing on well-established themes and terms in the vaccination decision literature while generating a list of themes that reflected participants’ reasons for vaccination intentions. This approach is also consistent with the flexibility of thematic analysis, [35, 36] which does not restrict the generation of themes to fit specific elements of a single framework.

The analysts independently reviewed a random subset of the data (i.e., 10%, 550 responses) to develop an initial set of codes using an inductive process (i.e., identifying emergent codes from the data). After reaching consensus on this initial set of codes, the analysts independently applied the codes to the random subset of responses. Codes were assigned to each individual response as applicable. More than one code was applied to each response if multiple reasons were listed as to why participants would or would not receive the COVID-19 vaccine. Responses that were not clear to the coder (e.g., “don’t know” or “none”) and/or responses that had multiple meanings (e.g., “I'm not stupid”, “safety”) were categorized as “Unclear”.

Per recommendations in the literature [37], intercoder reliability was calculated to measure initial agreement in the analysts’ coding for the random subset of responses and to refine the codebook for use in the full dataset, particularly for codes with lower reliability. Intercoder reliability was assessed using Cohen’s Kappa for each of the codes, along with a single reliability metric that averaged the Cohen’s Kappas across codes.

The analysts met to confirm that codes were applied consistently to the initial subset of data, to revise the codebook as needed by building upon or modifying existing codes, to finalize the codebook, and to reconcile any differences in coding through consensus. When no new codes emerged, coding saturation was met. The final codebook, including definitions and illustrative quotes, is included in the supplemental material (Table S1). Next, the analysts independently coded the remaining responses in the dataset (i.e., 5000 responses) using the final codebook. The qualitative analysis team (JRF, JR and ATF) reviewed the set of codes to identify themes in the data and thoughtfully considered all coding decisions. Drawing on guidance to collapse codes into themes based on shared concepts [35], higher-order themes were created when codes were conceptually related in at least one shared domain. Related codes were combined into higher level themes using both inductive terms drawn from the data and deductive terms drawn from the vaccination decision literature. This iterative, refining process allowed for meaningful interpretation of the data by using a manageable number of themes.

#### Integration of Qualitative and Quantitative Data

In the initial qualitative coding process, all 5,500 responses were assigned themes by the two coding analysts. To assess frequencies in the data, each theme was transformed into a separate, quantitative binary variable [38] and assigned a 1 if the theme was present in the open-ended reason and 0 if the theme was absent. All conflicts in assigned themes were resolved through consensus. The theme variables were then linked with the CVAC data (i.e., participants were assigned county-level barrier scores by ZIP Code).

#### Themes in COVID-19 Vaccination Intentions By County-Level Barriers

Frequencies of themes in COVID-19 vaccination intentions were assessed using descriptive statistics. Chi-square tests were used to examine whether the presence of theme in participants’ responses varied based on whether they lived in counties with low/medium or high/very high vaccination barriers.

#### Racial/Ethnic Differences in Themes in COVID-19 Vaccination Intentions by County-Level Barriers

Racial/ethnic differences were examined for the themes in COVID-19 vaccination intentions that varied by county-level barriers (i.e., chi-square test p-values < 0.10). A second set of chi-square tests were used to examine whether these distributions of open-ended reasons by county-level barriers varied across racial/ethnic groups.

All analyses were weighted to be nationally representative within each racial/ethnic group and were conducted in *R* version 4.2.1.

## Results

**Fig. 1:**
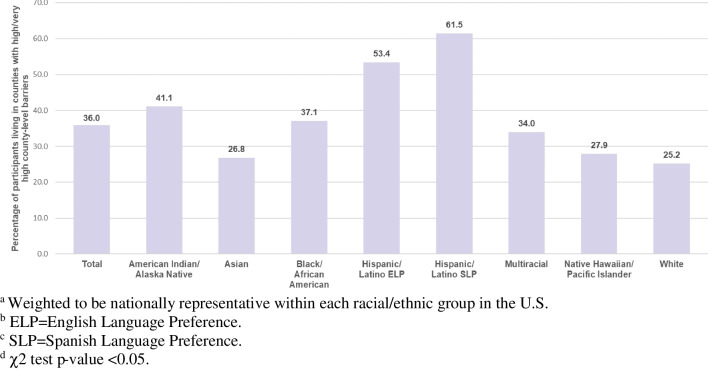
Participants (%) in counties with high/very high county-level barriers, total population and by racial/ethnic group. **a** Weighted to be nationally representative within each racial/ethnic group in the U.S. **b** ELP = English Language Preference. **c** SLP = Spanish Language Preference. **d** χ2 test p-value < 0.05

Selected study population characteristics, overall and stratified by racial/ethnic group, are presented in Table 1. County-level barriers, overall and stratified by racial/ethnic group, are presented in Fig. 1. Overall, 36% of participants lived in counties with high/very high vaccination barriers and these proportions varied by race/ethnicity (p-value  < 0.01, Fig. 1). For example, the prevalence of living in a county with high/very high vaccination barriers was 61.5% among Hispanic/Latino SLP adults and 53.4% among Hispanic/Latino ELP adults, and 25.2% among White adults. An in-depth discussion of racial/ethnic differences in county-level barriers has been discussed previously [22].

**Table 1:** Characteristics of the study population, overall and stratified by racial/ethnic group (weighted)

	Total (N = 5499)	AI/AN^c^ (n = 498)	Asian (n = 997)	Black/AA^d^ (n = 995)	Hispanic/Latino ELP^e^ (n = 495)	Hispanic/Latino SLP^f^ (n = 503)	Multi-racial (n = 499)	NH/PI^g^ (N = 499)	White (N = 992)
Age (Years), %									
18–34	34.7	35.3	34.0	33.4	42.2	35.5	47.2	36.8	25.2
35–49	26.7	22.9	29.5	23.9	26.7	36.4	24.7	34.4	21.0
50–64	23.8	26.2	23.0	26.8	13.5	24.5	17.9	22.0	28.9
65 and older	14.7	15.6	13.4	15.9	17.6	3.6	10.3	6.9	24.9
Gender, %									
Man	43.8	35.2	45.5	46.3	49.2	38.5	46.0	33.7	48.1
Woman	54.3	62.1	53.2	53.1	49.7	61.1	47.2	63.3	50.5
Non-binary	1.1	2.3	0.6	0.5	0.6	0.2	4.5	0.3	0.8
Transgender	0.3	0.3	0.2	0.1	0.2	0.2	0.3	2.2	0.0
Not listed	0.5	0.1	0.6	0.0	0.3	0.0	2.0	0.5	0.6
Education, %									
High school or less	39.7	42.4	23.6	42.2	53.1	67.1	32.0	45.9	32.4
Some college, 2-year college	33.0	44.4	23.8	38.0	31.1	21.0	37.9	37.9	33.5
4-year college	16.7	8.0	30.5	12.0	8.8	9.2	19.0	11.5	21.1
Post-graduate	10.6	5.1	22.1	7.7	7.1	2.7	11.1	4.7	13.1
Income, %									
Less than 20 K	29.7	42.8	18.3	41.3	29.4	32.1	24.7	38.0	20.4
20-49 K	30.9	29.8	25.7	30.2	35.5	48.7	27.8	26.6	29.7
50-100 K	25.1	20.1	28.6	19.5	24.8	15.3	33.0	25.9	30.2
100 K and over	14.3	7.3	27.4	9.0	10.3	3.9	14.6	9.5	19.6
Political Ideology, %									
Conservative	23.7	28.9	18.9	15.1	21.7	17.8	16.1	26.6	40.6
Liberal	29.6	22.6	33.0	31.8	33.8	26.5	42.4	19.2	26.0
Moderate	32.6	30.4	39.2	36.9	31.9	28.2	31.6	30.6	26.8
Not sure	14.1	18.1	8.9	16.2	12.7	27.6	9.9	23.6	6.6
Chronic Health Condition^h^, %									
High-Risk	41.9	50.3	33.3	48.4	37.5	31.1	41.4	41.7	47.6
Not high-risk	58.1	49.7	66.7	51.6	62.5	68.9	58.6	58.3	52.3
Health Insurance									
Covered	84.8	87.8	91.0	84.4	82.2	58.4	88.7	83.6	90.8
Not covered	15.2	12.2	9.0	15.6	17.8	41.6	11.3	16.4	9.2

^a^ Weighted to be nationally representative within each racial/ethnic group

^b^ All χ2 test p-values < 0.05

^c^ AI/AN = American Indian/Alaska Native

^d^ Black/AA = Black/African American

^e^ Hispanic/Latino ELP = Hispanic/Latino English Language Preference

^f^ Hispanic/Latino SLP = Hispanic/Latino Spanish Language Preference

^g^ NH/PI = Native Hawaiian/Pacific Islander

^h^ At the time of data collection, only adults with high-risk chronic health conditions were eligible to receive the COVID-19 vaccine. Given this eligibility, participants with high-risk chronic health conditions may have been more likely to have received at least one dose of the COVID-19 vaccine. Participants reported whether they had a chronic health condition by indicating whether a medical doctor or health professional ever told them they had a chronic health condition (i.e., checking all that apply from a list of chronic health conditions). Participants’ responses were coded as “high-risk” (1) or “not high-risk” (0) using the Centers for Disease Control (CDC) guidelines on medical conditions that increased the risk for severe illness due to COVID-19 (Kates J, Dawson L, Tolbert J: *The Next Phase of Vaccine Distribution: High-Risk Medical Conditions* from https://www.kff.org/policy-watch/the-next-phase-of-vaccine-distribution-high-risk-medical-conditions)

The average intercoder reliability of the initial, random subset of responses indicated substantial agreement per widely used criteria [39](Cohen’s Kappa: 0.67 ± 0.17).

### Themes in COVID-19 Vaccination Intentions

Consistent with recent literature [30], reasons why participants would receive the COVID-19 vaccine were characterized as “motivators” and reasons why participants would not receive the vaccine were characterized as “barriers.” Six themes were characterized as motivators and six themes were characterized as barriers. The twelve themes are described in detail below and listed in Table 2.

**Table 2:** Themes in participants’ reasons for COVID-19 vaccination intentions

Theme	Illustrative quote(s)
**Motivators of COVID-19 vaccination intentions**	
Desire to protect oneself	“I would get the vaccine in order to build up my immunity to Covid-19.”
Desire to protect close others	“para proteger a mi familia” [to protect my family]
Societal responsibility	“It’s my duty as a citizen to contribute to herd immunity...”
Desire to return to normal	“I want to resume my normal life and I want to travel”
Trust in science, healthcare, and/or government	“Science has backed these vaccines.”; “it's recommended by the CDC”
Generally positive/received vaccine	“I would, already did the first dose”
**Barriers to COVID-19 vaccination intentions**	
Concerns over access	“Unable to access location(s), wait in long lines.”; “depends on costs”
Concerns over side effects, safety, or development	“...I think it was rushed and I don’t feel safe getting it.”
Mistrust vaccines, vaccine researchers/manufacturers, healthcare, media, and/or institutions	“I do not trust the people who push this on us.”
Vaccine ineffective, unreliable, not needed	“I don’t take vaccines for diseases with a 99% survival rate.”
History of not vaccinating, negative attitudes about vaccines, religious reasons, desire for autonomy	“Never get the flu shot”; “Not worried. I'm religious”
Generally negative	“No, hell nah”

### Motivators

Three motivator themes related to different forms of protection. The first theme included participants who reported that the COVID-19 vaccine would increase their immunity and keep them safe from COVID-19 infection (*desire to protect oneself*). Secondly, participants reported that they would receive the COVID-19 vaccine to protect their family, friends, or loved ones (*desire to protect close others*). Lastly, some reported that receiving the COVID-19 vaccine would stop the spread of the COVID-19 virus and protect society in general, and in many cases, receipt of the vaccine was described as a collective responsibility to build herd immunity (*societal responsibility*).

Additional motivator themes included the desire for the pandemic to end and to return to “normal life”, including work and/or recreational activities (*desire to return to normal*). Others noted a theme of trust, including trust in science broadly or the efficacy of vaccines, trust in doctors or medical experts’ recommendations, and/or trust in government leaders or federal agencies (*trust in science, healthcare, and/or the government*). Finally, many participants reported generally positive comments about receiving the COVID-19 vaccine without specificity (e.g., “I definitely want the vaccine when it is available to me” or “I will get it”). These responses were characterized together with those reporting they had received and/or scheduled at least one dose of the COVID-19 vaccine (*generally positive/received vaccine*).

### Barriers

Barrier themes were characterized as reasons that negatively impacted participants’ COVID-19 vaccination intentions and uptake. These themes included structural barriers (e.g., systemic issues that limited participants’ ability to access the COVID-19 vaccine) as well as attitudinal barriers (e.g., beliefs or perceptions that influenced participants’ willingness to receive the COVID-19 vaccine) [40]. Structural barriers included participants’ concerns over whether they could access the COVID-19 vaccine, such as the ability to travel to vaccination sites, costs of the vaccine, availability of the vaccine, and/or time and effort needed to schedule and receive the vaccine (*concerns over access*).

Attitudinal barriers included participants’ concerns over potential side effects and/or harms of the COVID-19 vaccine, uncertainty about whether the COVID-19 was safe for use, and if the development of the COVID-19 vaccine was rushed (*concerns over vaccine side effects, safety, or development*). In addition, some participants reported mistrust of vaccines, vaccine researchers and manufacturers, healthcare, the media and/or other institutions such as the government and those in positions of power (*mistrust vaccines, vaccine researchers/manufacturers, healthcare, media, and/or institutions*). Others were complacent and discussed reasons that the COVID-19 vaccine was not necessary including notions that the COVID-19 vaccine was ineffective (e.g., the vaccine would be ineffective when new strains of the COVID-19 virus emerged), unreliable (e.g., “wait for bugs to be worked out”, “not sure it is reliable”), and/or that the vaccine was not needed due to low perceived risks of COVID-19 infection (*vaccine ineffective, unreliable, not needed*). Some participants also reported that they historically chose not to vaccinate, had negative attitudes about vaccines in general, that they chose not to vaccinate due to religious reasons, and/or they reported that choosing not to vaccinate was their choice (*history of not vaccinating, negative attitudes about vaccines, religious reasons, desire for autonomy*). Lastly, some participants reported generally negative comments about receiving the COVID-19 vaccine without specificity (e.g., “No hell nah”) (*generally negative*).

### Themes in COVID-19 Vaccination Intentions by County-Level Barriers

Overall, the most frequently reported motivator theme included the desire to protect oneself (26.0%). The most frequently reported barrier theme included concerns over to vaccine side effects, safety, or development (26.2%). Most themes in COVID-19 vaccination intentions had roughly equivalent prevalence in counties with low/medium versus high/very high barriers (Table 3). In five of the twelve themes, the prevalence varied across county-level barriers (p-values < 0.05).

**Table 3:** Themes (%) in reasons for COVID-19 vaccination intentions, total study population and by county-level barriers

	Total population (N = 5499)	Low/medium county-level barriers (n = 3520)	High/very high county-level barriers (n = 1979)	
Drivers of COVID-19 vaccination intentions, %				p-value
Desire to protect oneself	26.0	25.8	26.4	0.63
Generally positive/received vaccine	10.7	11.1	10.2	0.32
Societal responsibility	9.1	9.8	7.7	0.01
Desire to return to normal	6.9	8.1	4.7	< 0.01
Trust in science, healthcare, and/or government	6.8	7.7	5.1	< 0.01
Desire to protect close others	6.7	6.4	7.3	0.22
Barriers to COVID-19 vaccination intentions, %				
Concerns over side effects, safety, or development	26.2	25.3	27.9	0.04
Vaccine ineffective, unreliable, not needed	8.4	8.3	8.6	0.74
Mistrust vaccines, vaccine researchers/manufacturers, healthcare, media, and/or institutions	7.9	7.6	8.4	0.26
Generally negative	3.7	3.8	3.6	0.71
Concerns over access	2.5	1.9	3.6	< 0.01
History of not vaccinating, negative attitudes about vaccines, religious reasons, desire for autonomy	2.9	3.1	2.6	0.26

^a^ Weighted to be nationally representative within each racial/ethnic group in the U.S

The motivator themes that varied across county-level barriers included societal responsibility, a desire to return to normal, and trust in science, healthcare and/or the government (Table 3). The prevalence of these motivator themes were higher in counties with low/medium (versus high/very high) vaccination barriers (i.e., societal responsibility: 9.8% versus 7.7%; desire to return to normal: 8.1% versus 4.7%; trust in science, healthcare, and/or the government: 7.7% versus 5.1%). The barrier themes that varied across county-level barriers included concerns over access and concerns over vaccine side effects, safety or development. In contrast to the motivator themes, the prevalence of these barrier themes were lower in counties with low/medium (versus high/very high) barriers (i.e., concerns over access: 1.9% versus 3.6%; concerns over vaccine side effects, safety or development: 25.3% versus 27.9%). No other differences across county-level barriers were observed for the remaining themes.

### Racial/Ethnic differences in Themes in COVID-19 Vaccination Intentions by County-Level Barriers

**Fig. 2:**
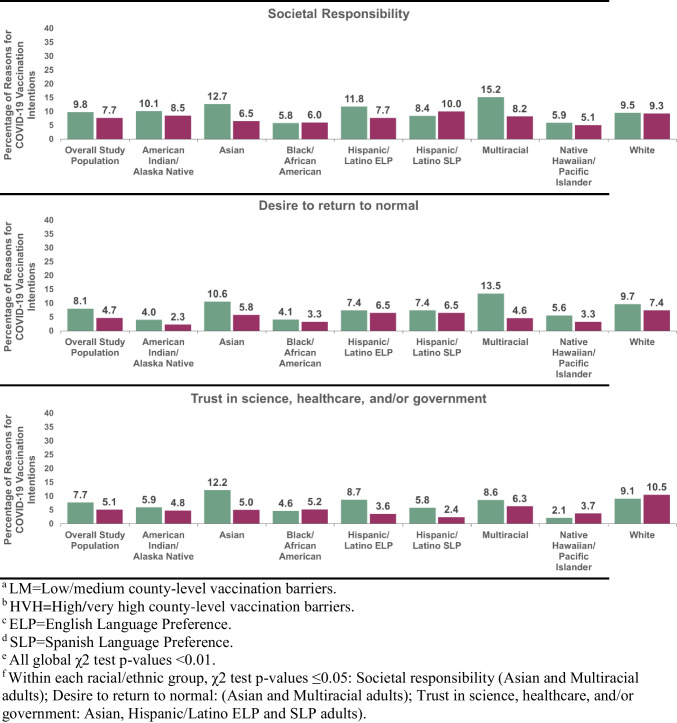
Motivator themes in reasons for COVID-19 vaccination intentions, stratified by county-level barriers and racial/ethnic group. **a** LM = Low/medium county-level vaccination barriers. **b** HVH = High/very high county-level vaccination. **c** ELP = English Language Preference. **d** SLP = Spanish Language Preference. **e** All global χ2 test p-values < 0.01. **f** Within each racial/ethnic group, χ2 test p-values ≤ 0.05: Societal responsibility (Asian and Multiracial adults); Desire to return to normal: (Asian and Multiracial adults); Trust in science, healthcare, and/or government: Asian, Hispanic/Latino ELP and SLP adults)

**Fig. 3:**
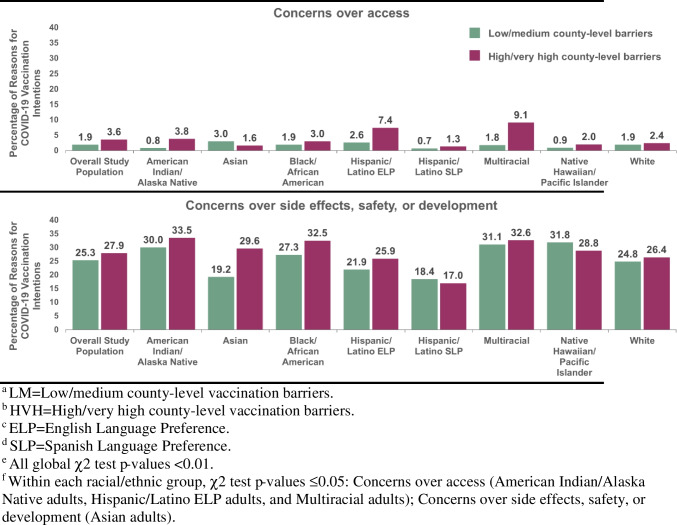
Barrier themes in reasons for COVID-19 vaccination intentions, stratified by county-level barriers and racial/ethnic group. **a** LM = Low/medium county-level vaccination barriers. **b** HVH = High/very high county-level vaccination. **c** ELP = English Language Preference. **d** SLP = Spanish Language Preference. **e** All global χ2 test p-values < 0.01. **f** Within each racial/ethnic group, χ2 test p-values ≤ 0.05: Concerns over access (American Indian/Alaska Native adults, Hispanic/Latino ELP adults, and Multiracial adults); Concerns over side effects, safety, or development (Asian adults)

Racial/ethnic differences were examined in the five themes that varied by county-level barriers. Global chi-square tests (p-values < 0.05) revealed that the distribution of all five themes stratified by county-level barriers differed across racial/ethnic groups (Figs. 2&3). Within motivator themes, the prevalence of societal responsibility and desire to return to normal themes varied by county-level barriers (p-values < 0.05) among Asian adults and Multiracial adults (Fig. 2). The prevalence of trust in science, healthcare, and/or the government theme varied by county-level barriers (p < 0.05) among Asian adults, Hispanic/Latino ELP adults, and Hispanic/Latino SLP adults. Consistent with the trends in the total population, in each of these racial/ethnic groups, the prevalence of the three motivator themes were higher in counties with low/medium (versus high/very high) barriers (societal responsibility: 12.7% versus 6.5% (Asian), 15.2% versus 8.2% (Multiracial); desire to return to normal: 10.6% versus 5.8% (Asian), 13.5% versus 4.6% (Multiracial); trust in science, healthcare, and/or the government: 12.2% versus 5.0% (Asian), 8.7% versus 3.6% (Hispanic/Latino ELP), 5.8% versus 2.4% (Hispanic/Latino SLP)).

Within barrier themes, the prevalence of concerns over access varied by county-level barriers (p-value < 0.05) among American Indian/Alaska Native, Hispanic/Latino ELP, and Multiracial adults (Fig. 3). Consistent with the trend in the total population, the prevalence of concerns over access were lower in counties with low/medium (versus high/very high) barriers (0.8% versus 3.8% (American Indian/Alaska Native), 2.6% versus 7.4% (Hispanic/Latino ELP), 1.8% versus 9.1% (Multiracial)). Lastly, the prevalence of concerns over vaccine side effects, safety, or development only varied by county-level barriers (p < 0.05) among Asian adults. Among Asian adults, there was a lower prevalence of concerns over vaccine side effects, safety, or development in counties with low/medium (versus high/very high) barriers (19.2% versus 29.6%). In all other racial/ethnic groups, the prevalence of concerns over vaccine side effects, safety, or development was generally high (> 20%) and roughly equivalent across county-level barriers.

## Discussion

The present study identified themes in open-ended reasons for COVID-19 vaccination intentions among a large, diverse population of adults living in the U.S. Although themes in participants’ responses reflected motivators and barriers to COVID-19 vaccination intentions that were largely consistent with recent literature [4, 5, 23, 30, 40], this work makes an important contribution to health behavior and health services research by examining how these themes varied by county-level barriers using a comprehensive measure that incorporated social vulnerabilities, healthcare access barriers and healthcare system resources. This study also assessed how reasons for COVID-19 vaccination intentions varied by county-level barriers across racial/ethnic groups. These findings may suggest promising opportunities to further explore why structural factors may differentially impact certain motivators and barriers to COVID-19 vaccination intentions across different racial/ethnic groups. In addition, this study allows for the identification of more intervention targets by geographical location where disparities exist in vaccine uptake [41].

Participants reported that they would receive the vaccine for protection (for themselves, their loved ones, or society in general), to return to normal, because they trusted science, healthcare, and/or the government, or they provided generally positive, non-specific comments about the vaccine. By contrast, participants reported themes in barriers that included structural barriers and attitudinal barriers, or they reported generally negative, non-specific comments about the COVID-19 vaccine.

The present study found that the prevalence of some themes in COVID-19 vaccination intentions varied based on participants’ county-level barriers. Three motivator themes (societal responsibility, a desire to return to normal, and trust in science, healthcare, or the government) were more frequently reported by participants living in counties with low/medium (versus high/very high) barriers. These findings may suggest that residents of counties with lower vaccination barriers were less concerned about the potential harms of the COVID-19 vaccine and therefore, residents were able to focus on vaccination benefits, such as allowing them to return to normal activities. In addition, based on the CVAC index, residents in counties with lower vaccination barriers had greater access and exposure to vaccine information (e.g., role of vaccine in herd immunity) and healthcare system resources which may have increased their confidence in the vaccine. Given that many other factors such as education and income vary by ZIP Code [42] and have been previously associated with willingness to receive the COVID-19 vaccine [43], the distribution of economic and social determinants of health across U.S. counties likely influenced the distribution of motivator themes in the present data.

Conversely, two barrier themes (concerns over access and concerns over vaccine side effects, safety or development) were more frequently reported by participants living in counties with high/very high (versus low/medium) barriers. Participants living in counties with higher vaccination barriers may have developed concerns about COVID-19 vaccination that outweighed all other considerations. It is possible, for example, that these counties have a longstanding history of having limited access to healthcare information (e.g., vaccine benefits) and resources such as providers who are a preferred information source. Previous studies reported that lack of convenience (e.g., access, costs, availability) of the vaccine contributes to higher levels of vaccine hesitancy, which could reflect concerns of those living in counties with higher vaccination barriers.

 The present study findings also suggested that county-level barriers had a larger role in determining reasons for COVID-19 vaccination intentions for some racial/ethnic groups compared to others. The motivator themes varied by county-level barriers among Asian adults, Multiracial adults, Hispanic/Latino ELP adults, and American Indian/Alaska Native adults. Many factors could explain these differences. For example, collectivistic values, which are generally high among many Asian and Hispanic/Latino cultures [44] have been previously linked to feelings of collective responsibility and trust in vaccines [45–47]. Perhaps, in counties where residents had fewer concerns about healthcare access and greater exposure to information and providers, collectivistic values emerged as important considerations when making vaccination decisions. Within barrier themes, this study found that among American Indian/Alaska Native, Hispanic/Latino ELP, and Multiracial adults, concerns over access were more prevalent in counties with higher barriers. These findings could suggest the need to further explore whether concerns over access were particularly influenced by county barriers in American Indian/Alaska Native, Hispanic/Latino ELP, and Multiracial communities. In addition, concerns over vaccine side effects, safety, and development were generally high among most racial/ethnic groups. In both barrier themes (concerns over access and vaccine side effects, safety, and development), the present study findings may relate to well-documented evidence of structural racism that contributed to vaccine hesitancy in the early stages of the COVID-19 pandemic [34, 48–50]. For example, structural factors such as the complexity of the vaccine scheduling system, language requirements, perceived barriers related to immigrant status, travel to vaccination sites, computer/internet access, and/or lack of accessible and trusted clinics were identified as system-level barriers that contributed to COVID-19 vaccination-related health disparities among Black, Hispanic/Latino, and older Asian communities [34, 49]. It is possible that in many cases, structural racism contributed to concerns over access, and/or concerns over vaccine side effects, safety, and development at the time of study, regardless of the specific county-level barriers measured by the CVAC.

Lastly, given the qualitative coding of reasons for COVID-19 vaccination intentions, the present study assessed racial/ethnic group differences in the prevalence of reasons using descriptive statistics rather than estimating prevalence using adjusted statistical models. Observed differences may reflect sociodemographic characteristics that varied across racial/ethnic groups. For example, Asian adults in the sample had higher proportions of participants with more education and higher incomes, whereas Multiracial adults had higher proportions of participants who were younger (i.e., aged 18–34 years) and Liberal. Themes in COVID-19 intentions may have been shaped by both individual and county-level factors.

Limitations should be considered. The survey was conducted online and those who participated had internet access. Internet access has been associated with higher income [51] and lack of internet access was a barrier to COVID-19 vaccination in previous studies [52, 53]. These factors may have influenced participants’ reasons why they would or would not receive the vaccine. In addition, the survey was administered in English and Spanish languages, thereby limiting generalizability to non-English and non-Spanish speakers living in the U.S. English language proficiency has also been previously identified as a barrier to COVID-19 vaccination across all racial/ethnic groups [54] which was not captured in the present study. Additionally, it is also possible that the study population was affected by non-response bias, particularly because the study was government-sponsored and those who did not trust the government may have been less likely to participate. The cross-sectional survey also limited the ability to make causal claims about county-level barriers and reasons for COVID-19 vaccination. With respect to measurement, the CVAC index included limitations in its development (e.g., missing data required imputing data at the median county levels, and the index has yet to be fully validated). The open-ended structure of the reasons question may have also introduced some potential limitations. Roughly 9% of responses were categorized as unclear, which was often due to brevity, and participants may not have listed all reasons for their vaccination intentions. Lastly, the qualitative themes in the data captured themes in the responses, yet there were likely situations when themes were highly related. For example, some responses, such as believing the vaccine to be unreliable could have also been interpreted as mistrusting the vaccine. To the extent possible, codes were applied based on participants’ words and the analysts attempted to limit overinterpreting responses. However, recognizing the potential for overlap may suggest a need to cautiously interpret quantitative differences in prevalence of themes, particularly in situations when differences were small.

Strengths of the present study included the large, diverse study population across seven racial/ethnic groups in the U.S. This included substantial representation from American Indian/Alaska Native, Native Hawaiian/Pacific Islander, and Multiracial adults who are often underrepresented in national surveys. This study was also the first study to examine open-ended reasons for COVID-19 vaccination intentions across county-level barriers in the CVAC index. The CVAC index is a newly developed tool that extends literature on social vulnerabilities and health services use by including healthcare access barriers and limited healthcare system resources. These findings are especially important given limited evidence on the association between structural factors and individuals’ reasons for COVID-19 vaccination intentions. The open-ended nature of the question asking participants to list reasons for their COVID-19 vaccination intentions also likely captured the reasons that were most important to participants. Lastly, this study included both reasons why participants would and would not vaccinate, whereas previous literature focused on reasons participants would not vaccinate. Therefore, this study makes an important contribution to the literature on motivators of health vaccination behavior.

## Conclusions

Findings from the present study suggest that U.S. adults’ vaccination behaviors, particularly in the earlier stages of the COVID-19 pandemic, were driven by individual-level considerations (e.g., attitudes and beliefs about the vaccine, healthcare, and protection of oneself and others) as well as system-level factors (e.g., concerns over access, convenience, and historical mistrust in the healthcare system). Moreover, the prevalence of certain themes for COVID-19 vaccination intentions varied across county-level barriers and racial/ethnic groups. These findings emphasize the importance of social determinants, and may also suggest the impact of structural racism in shaping how U.S. adults think about health services and newly developed vaccines. Additionally, the present study may be especially useful in designing health communication for future pandemics. In counties with lower vaccination barriers, vaccination campaigns could consider emphasizing motivators of vaccination such as the ability to use vaccines to protect oneself, loved ones, and the community, to return to normal activities, and the efficacy of the vaccine in reducing COVID-19 infection rates throughout the country. In counties with higher vaccination barriers, vaccination campaigns would likely need to address concerns about vaccine side effects, access, and development, mistrust in the vaccine and other institutions, beliefs that the COVID-19 vaccine was ineffective or unreliable, and general negative attitudes about vaccines. Strategies would need to focus on communicating this information in a way that is clear, equitable, and driven by the needs and concerns of the community. The use of tailored health communication based on large, nationally representative surveys capturing individuals’ reasons for health behavior will continue to be an important factor in the ongoing management of the COVID-19 virus and public health responses to future pandemics.

## Supplementary Information

Below is the link to the electronic supplementary material.

Supplementary file1 (PDF 202 KB)

## Data Availability

The dataset used during the current study is available from the corresponding author on reasonable request.

## Declarations

### Disclaimers

The contents and views in this manuscript are those of the authors and should not be construed to represent the views of the National Institute on Minority Health and Health Disparities, National Institutes of Health, or the Department of Health and Human Services. JCE is an education consultant for MERCK, ChromaHealth, and Foundation for Sarcoidosis Research.
