# Supplementary material for: Motivators and Barriers to COVID-19 Vaccination Intentions Across U.S. County-Level Barriers in the COVID-19 Vaccine Coverage Index
Source: J Racial Ethn Health Disparities. 2024 Aug 2;12(5):2882–95. doi: 10.1007/s40615-024-02096-9 (PMC11866401; doi:10.1007/s40615-024-02096-9)
Supplement: Supplementary file 1 — Supplementary file1 (PDF 202 KB) [file 40615_2024_2096_MOESM1_ESM.pdf]

Motivators and Barriers to COVID-19 Vaccination Intentions Across U.S. County-Level Barriers in the COVID-19 Vaccine Coverage Index
Supplemental Materials

Table S1. Codebook of themes for COVID-19 vaccination intentions

Code/Theme	Description	Illustrative Quotes
**Motivators of COVID-19 vaccination intentions**		
**Desire to protect oneself**	**Response mentions:**<br>- Vaccine offers safety/protection/immunity, avoiding becoming infected from COVID-19, or personal health.<br>- Needing vaccine due to health status (age, immunocompromised, etc).<br>- COVID-19 threat (e.g. COVID is dangerous, COVID is serious, or fear/being scared of COVID)	"To be protected"<br>"I would get the vaccine in order to build up my immunity to Covid-19."<br>"to avoid becoming infected with COVID-19"<br>"I am high risk"<br>"Because I am 81 years old and immunocompromised"
**Desire to protect close others**	**Response mentions:**<br>- Protecting one's friends, family or loved ones from the COVID-19 virus.	"to stay safe and healthy for friends and family we all should do the same to make sur it dose not keep spreding to our love ones"<br>"para proteger a mi familia" [to protect my family]<br>"To make sure I can safely visit friends and family going forward"
**Societal responsibility**	**Response mentions:**<br>- Vaccination as a social responsibility, benefits of vaccinating for society overall, COVID-19 vaccination is the responsible thing to do, protecting vulnerable populations, protecting other people in general or preventing the spread of the virus to others, and/or mentions herd immunity.	"Ensuring that we can reach herd immunity by doing my part and helping others."<br>"I am sure that it is necessary for all of us to do our part to help contain this disease and protect the most vulnerable and one of the best way to do it is by vaccinating ourselves."<br>"I think it's the right thing to do"
**Desire to return to normalcy**	**Response mentions:**<br>- Ending the pandemic, going "back", returning to normalcy/normal activities (e.g., concerts, travel, work)<br>- Will vaccinate if required to return to work or engage in certain activities (e.g., frontline health force, university, travel, something else)	" If getting a vaccine can prevent the transmission of covid, then we need to take the vaccine to get back to normal faster."<br>"It is required by my job to get the vaccine"<br>"I want to resume my normal life and I want to travel"
**Trust in science, healthcare and/or the government**	**Response mentions:**<br>- Trusting in science, research, and or evidence.<br>- Confidence in effectiveness of the vaccine, limited side effects, and/or confidence in the vaccine safety, trusting personal doctor's recommendation, health care providers, pharmaceutical companies, government or public agencies.	"Science has backed these vaccines."<br>"Because the vaccine is safe"<br>"I trust medical professionals"<br>"it's recommended by the CDC"
**Generally positive/received vaccine**	**Response mentions:**<br>- Already made decision to receive the vaccine (e.g., received it, have appointment scheduled, will schedule an appointment as soon as possible/is their turn).<br>- General positive comments about COVID-19 vaccine without details.	"I would, already did the first dose"<br>"I will get a vaccine when it becomes available to the general public after those who are more vulnerable than myself."
**Barriers to COVID-19 vaccination**		
**Concerns over access**	**Response mentions:**<br>- Concerns about being able to access the vaccine<br>- Concerns over availability of the vaccine (for self or others)<br>- Concerns about traveling to the vaccination sites<br>- Concerns over the cost of the vaccine<br>- Concerns over convenience (scheduling appointment, long lines)	"I will only get if it is convenient. I will not stand in long lines."<br>"Unable to access location(s), wait in long lines."<br>"depends on costs"<br>"It's too complicated to get one now"<br>"I will get it when the process is easier. Like when it's available at pharmacies or at the free clinic I go to."

Motivators and Barriers to COVID-19 Vaccination Intentions Across U.S. County-Level Barriers in the COVID-19 Vaccine Coverage Index Supplemental Materials

Code/Theme	Description	Illustrative Quotes
**Concerns over side effects, safety, or development**	**Response mentions:**<br>- Concern over safety, side effects, allergic reactions, interference with other medical conditions (including pregnancy), contents of the vaccine (e.g., mRNA, alters genes, "poison", foreign contents, chip in vaccine).<br>- Concerns about the newness of the vaccine, speed of its development, lack of FDA approval, concerns about scientists/research.<br>- Waiting for information or evidence about long-term effects of vaccine.	"Don't want poison in my body"<br>"I don't feel comfortable getting it. I think it was rushed and I don't feel safe getting it."<br>"Heard someone died from the vaccine"<br>"Don't trust the studies because it was a rushed process"<br>"I want to wait and see how the first several million people respond to the vaccine."<br>"Need more testing"
**Mistrust vaccines, vaccine researchers/ manufacturers, healthcare, media, and/or institutions**	**Response mentions:**<br>- Not trusting the vaccine, doctors, health care providers, the health system, and/or pharmaceutical companies<br>- Mistrust in government, public agencies, the media (e.g., fake news), they are tracking us, not trusting Democrats or other political motives, other power entities (e.g. Bill Gates, elitists)	"I don't trust it [the vaccine]"<br>""I do not trust the people who push this on us."<br>"For one thing I do not trust doctors fully. As a woman and as a Filipina woman if I get a white doctor they do not listen to me, they do not take me seriously. I trust in facts and science and most doctors are okay, but many are elitist and racist."<br>"It`s a sham used by biden and the government to put chips in people to run our lives"<br>"As a black person living in America I honestly don't trust anything. The government always uses black people as their guinea pigs and test dummies. That sh*t is f*ck*ng sickening. The government is trying to basically bribe us to take the vaccine by sho wing us black public figures taking the vaccine thinking that we're going to follow suit. Tyler Perry nor T.I are the voice or spokesperson for black people and it's very insulting to think that."<br>"I do not believe the testing numbers are accurate. I believe Democratic government is behind all of this by inflating the numbers and causing mass hysteria. The media also plays a part in all of this. Personal experience with a family member that passed away from a stroke but hospital wanted to say Covid which was NOT the case. I just don't trust any of this!!"
**Vaccine ineffective, unreliable, not needed**	**Response mentions:**<br>- Vaccine will be ineffective or unreliable at protecting against COVID-19 and variants<br>- COVID-19 vaccine is unnecessary (e.g., COVID-19 is not serious or because they already had the virus)<br>- Low risk perceptions (i.e. low severity and/or susceptibility).	"I'll either get COVID or not. Either way, vaccinations always make me sick. I'll take my chances"<br>"I don't take vaccines for diseases with a 99% survival rate."<br>"Don't currently have covid"
**History of not vaccinating, negative attitudes about vaccines, religious reasons, desire for autonomy**	**Response mentions:**<br>- Not getting flu vaccine, not liking needles, or that they do not take/like any vaccines or medicines.<br>- Not getting vaccine for religious reasons.<br>- Freedom of choice/my decision	"I'm anti vaccine"<br>"Never get the flu shot"<br>"God made my natural immunity and I do not take vaccines"<br>"Religion thing dont believe in vaccines only certain ones"<br>"por miedo a las agujas" [for fear of needles]<br>"My choice"
**Generally negative**	**Response mentions:**<br>- General negative comments without additional details.	"No, hell nah"<br>"Suspicious"
**Unclear response**	Respondents' comments are unclear, blank, "don't know", "none", or response could have multiple meanings (e.g., I'm not stupid, safety)	*A missing response*<br>"Don't know"
